# Impact of pre-operative weight loss on non-alcoholic fatty liver disease histopathology and insulin resistance in individuals undergoing bariatric surgery: a propensity matched cross-sectional comparison

**DOI:** 10.1590/1516-3180.2022.0663.R1.24042023

**Published:** 2023-06-09

**Authors:** Fábio Henrique Ribeiro Maldonado, Paulo Ferreira Mega, Carlos Wustemberg Germano, Luana Leite Castilho Dias, Guilherme Hoverter Callejas, Martinho Antonio Gestic, Murillo Pimentel Utrini, Felipe David Mendonça Chaim, Francisco Callejas-Neto, Elinton Adami Chaim, Everton Cazzo

**Affiliations:** I MD. Resident Physician, Department of Surgery, Campinas Medical Center (CMC), Campinas (SP), Brazil.; II MD. Resident Physician, Department of Surgery, Universidade Estadual de Campinas (UNICAMP), Campinas (SP), Brazil.; III MD. Resident Physician, Department of Surgery, Universidade Estadual de Campinas (UNICAMP), Campinas (SP), Brazil.; IV BM. Undergraduate Student, Faculty of Medicine, Pontíficia Universidade Católica de Campinas (PUCCAMP), Campinas (SP), Brazil.; V MD, MSc. Resident Physician, Department of Surgery, Universidade Estadual de Campinas (UNICAMP), Campinas (SP), Brazil.; VI MD, MSc. Assistant Lecturer, Department of Surgery, Universidade Estadual de Campinas (UNICAMP), Campinas (SP), Brazil.; VII MD. Assistant Lecturer, Department of Surgery, Universidade Estadual de Campinas (UNICAMP), Campinas (SP), Brazil.; VIII MD, PhD. Assistant Lecturer, Department of Surgery, Universidade Estadual de Campinas (UNICAMP), Campinas (SP), Brazil.; IX MD, MSc. Assistant Professor, Department of Surgery, Universidade Estadual de Campinas (UNICAMP), Campinas (SP), Brazil.; X MD, PhD. Full Professor, Department of Surgery, Universidade Estadual de Campinas (UNICAMP), Campinas (SP), Brazil.; XI MD, PhD. Associate Professor Department of Surgery, Universidade Estadual de Campinas (UNICAMP), Campinas (SP), Brazil.

**Keywords:** Non-alcoholic fatty liver disease, Obesity, Bariatric surgery, Weight loss, Insulin resistance, Liver steatosis, Non-alcoholic steatohepatitis, Liver fibrosis, Hepatocellular ballooning, Hepatic lobular inflammation, Hepatic portal inflammation

## Abstract

**BACKGROUND:**

The effect of weight loss (WL) on histopathological aspects of non-alcoholic fatty liver disease (NAFLD) may provide further insights into the dynamics of hepatic recovery after WL.

**OBJECTIVE:**

To analyze the effects of pre-operative WL on insulin resistance- and NAFLD-related histology in individuals undergoing bariatric surgery (BS) with or without pre-operative WL.

**DESIGN AND SETTING:**

A matched cross-sectional study was conducted at a public university hospital and a private clinic in Campinas, Brazil.

**METHODS:**

An analytical, observational, cross-sectional study was conducted using prospectively collected databases of individuals who underwent BS and liver biopsy at either a public tertiary university hospital (with pre-operative WL) or a private clinic (without pre-operative WL). Random electronic matching by gender, age, and body mass index (BMI) was performed and two paired groups of 24 individuals each were selected.

**RESULTS:**

Of the 48 participants, 75% were female. The mean age was 37.4 ± 9.6. The mean BMI was 38.9 ± 2.6 kg/m^2^. Fibrosis was the most common histopathological abnormality (91.7%). Glucose was significantly lower in the WL group (92 ± 19.1 versus 111.8 ± 35.4 mg/dL; P = 0.02). Significantly lower frequencies of macrovesicular steatosis (58.3% versus 95.8%; P = 0.004), microvesicular steatosis (12.5% versus 87.5%; P < 0.001), and portal inflammation (50% versus 87.5%; P = 0.011) were observed in the WL group.

**CONCLUSION:**

Pre-operative WL was significantly associated with lower frequencies of macro- and mi- crovesicular steatosis, portal inflammation, and lower glycemia, indicating an association between the recent trajectory of body weight and histological aspects of NAFLD.

## INTRODUCTION

Non-alcoholic fatty liver disease (NAFLD) is an abnormal accumulation of fat in the liver in the absence of excessive alcohol drinking and/or any other secondary cause.^1–3^ Alongside the obesity epidemic, it has been increasingly detected over the last few decades and has now been acknowledged as a source of public health concern. NAFLD is currently considered the most common liver disease worldwide. It comprises a wide spectrum of histological features, ranging from mild steatosis to severe forms of fibrosis, steatohepatitis, cirrhosis, and hepatocellular car- cinoma.^1,2,4^ NAFLD and non-alcoholic steatohepatitis (NASH) occur in approximately 20–30% and 2–3% of the general Western population, respectively.^4,5^ Several studies have reported high rates of NAFLD among individuals who undergo bariatric surgery (BS). Within this group, the prevalence of liver fibrosis reportedly ranges from 6% to 74.4% and that of steatohepatitis from 26% to 55%.^6–9^

Weight loss (WL) is a key factor in treating NAFLD. Studies involving individuals undergoing BS have indicated that some conditions, such as steatosis and steatohepatitis, are more prone to weight changes, while fibrosis appears to be more refractory. However, most of these studies were based on non-invasive assessment methods (non-invasive laboratory markers and other imaging methods). Studies based on paired biopsies were usually based on the comparison of generic elementary histological variables (steatosis, steatohepatitis, and fibrosis) without differentiating between specific profiles of steatosis or inflammation, for example.^10–13^

A detailed characterization of the impact of WL on the histopathological aspects related to NAFLD and markers of insulin resistance (IR) may provide further insights into the pathophysiology related to the treatment of the disease and the dynamics of hepatic histological recovery after WL.

## OBJECTIVE

This study aimed to analyze the effects of pre-operative WL on the biochemical parameters of IR and NAFLD-related histological findings in obese individuals undergoing BS by comparing groups that either underwent pre-operative loss or did not, matched by gender, age, and body mass index (BMI).

## METHODS

### Study design

An analytical, observational, cross-sectional study was conducted based on data from prospectively collected databases of individuals who underwent BS at either a public tertiary university hospital or private clinic. Individuals from the public hospital underwent surgery in 2019, and those from the private clinic in 2022. In public hospitals, all individuals undergo a mandatory pre-operative WL program before surgery, which is not performed in a private facility.

The study protocol was analyzed and approved by the Ethics Committee of the Hospital de Clínicas, Universidade Estadual de Campinas (HC-UNICAMP) on April 4, 2022, under the opinion CAAE:55719322.9.0000.5404. All the participants provided informed consent.

### Study population and matching procedure

The study protocol included individuals of any gender aged 18–70 who underwent BS, as indicated by the National Institutes of Health criteria. Exclusion criteria included individuals belonging to vulnerable groups (underaged and/or mentally/ intellectually disabled), with past or current unrelated liver disease, previous or current history of alcohol use, previous or current cholestasis, previous liver surgery, previous or current use of hepatotoxic medications, chronic viral hepatitis, or incomplete medical records.

We identified 113 individuals who met the criteria at a public hospital (who underwent pre-operative WL) and 29 individuals at a private facility (who did not undergo pre-operative WL). A random electronic matching of the databases was performed using the Statistic Analysis System (SAS) software, via the “PSMATCH” procedure, using the selected variables age, gender, and BMI in a 1:1 ratio; two paired groups of 24 individuals each were selected. The BS procedures performed by the study participants were Roux-en-Y gastric bypass (n = 35) or sleeve gastrectomy (n = 13).

### Pre-operative weight loss

All individuals who underwent surgery at the public hospital were included in a multidisciplinary group that performed pre-operative preparation for the procedure. It included weekly consultations with a multidisciplinary team (surgeon, nurse, psychologist, and dietitian), during which they received general guidance, psychological counseling, and dietary prescriptions. Individuals undergo BS after approximately 30–60 days when they achieve a 10–20% WL or when the minimum BMI is close to 35 kg/m^2^. No medication for WL was prescribed.^14^

Individuals from private clinics undergo standardized multidisciplinary pre-operative assessments before BS, and there are no specific or systematic recommendations for pre-operative WL.

### Stratification by groups

Participants were categorized into two groups according to pre-operative WL in relation to baseline weight: (I) individuals who underwent pre-operative WL and (II) individuals without pre-operative WL.

### Liver biopsy technique

All patients underwent systematic wedge liver biopsy during BS, following a standardized method. An approximately 2-cm fragment was extracted from segment III or IV of the liver at the end of the surgical procedure. Histopathological analyses of both groups were performed and reviewed under the supervision of the same pathology team.

### Variables

The following demographic and anthropometric data were compared between groups: age, gender, BMI at the time of surgery (time of liver biopsy). For individuals who underwent pre-oper- ative WL, the baseline BMI and percentage of total WL (%TWL) were also assessed.

Biochemical examinations included aspartate aminotransferase, alanine aminotransferase, glucose, and insulin levels. Homeostasis model assessment – IR (HOMA-IR) was calculated according to Matthews’ formula.^15^

Histopathological analyses were performed by hematoxylin and eosin staining. The presence or absence of the following histopathological variables were considered: macrovesicular steatosis, microvesicular steatosis, lobular inflammation, portal inflammation, fibrosis, iron overload, and hepatocellular ballooning.

### Statistical analysis

Chi-square and Fisher’s exact tests were used to compare proportions. The Mann-Whitney U test was used to compare continuous variables between independent groups. The level of significance for the statistical tests was set at 5% (P < 0.05). To perform the analyses, we employed software SAS System for Windows (Statistic Analysis System), version 9.2; SAS Institute Inc., 2002–2008, Cary, North Carolina, United States.

## RESULTS

Of the 48 participants, 75% were female. The mean age was 37.4 ± 9.6. The mean baseline BMI of the pre-operative loss group was 47.7 ± 4.8 kg/m^2^; its mean pre-operative %TWL was 17.9 ± 6.2%. The overall mean BMI of the study population at the time of surgery was 38.9 ± 2.6 kg/m^2^. The most common histopathological abnormalities observed were fibrosis (91.7%) and macrovesicular steatosis (77.1%). Table 1 provides a complete description of the demographic, anthropometric, biochemical, and histopathological characteristics of study participants.

**Table 1:** Demographic, anthropometric, biochemical, and histopathological characteristics of the overall study population

n		48
**Age (years)**		37.4 ± 9.6
**Gender**		
	Male	12 (25%)
	Female	36 (75%)
**Baseline BMI (kg/m** ^2^) **(n = 24)**		47.7 ± 4.8
**BMI at surgery (kg/m** ^2^)		38.9 ± 2.6
**%TWL (n = 24)**		17.9 ± 6.2
**AST (IU/L)**		24.9 ± 11.6
**ALT (IU/L)**		32.5 ± 22.6
**Glucose (mg/dL)**		99.6 ± 27.3
**Insulin (**μ**U/mL)**		25 ± 13
**HOMA-IR**		6.2 ± 3.6
**Macrovesicular steatosis – n (%)**		37 (77.1%)
**Microvesicular steatosis – n (%)**		24 (50%)
**Lobular inflammation – n (%)**		32 (66.7%)
**Portal inflammation – n (%)**		33 (68.8%)
**Fibrosis – n (%)**		44 (91.7%)
**Hepatocellular ballooning – n (%)**		34 (70.8%)
**Iron overload – n (%)**		24 (50%)

n = number of individuals; BMI = body mass index; AST = aspartate aminotransferase; ALT = alanine aminotransferase; HOMA-IR = homeostasis model assessment- insulin resistance.

The groups did not differ in age, gender, or BMI at the time of liver biopsy or BS. Regarding biochemical variables, blood glucose was significantly lower in the pre-operative WL group (92 ± 19.1 versus 111.8 ± 35.4 mg/dL; P = 0.02). A complete comparison of the demographic, anthropometric, and biochemical variables is presented in Table 2.

**Table 2:** Comparison of demographic, anthropometric, biochemical, and histopathological variables between groups with or without pre-operative weight loss

n		Pre-op WL	No pre-op WL	P value
		24	24	NA
**Age (years)**		37.5 ± 8.5	37.2 ± 10.8	0.89
**Gender**				
	Male	6 (25%)	6 (25%)	1.00
	Female	18 (75%)	18 (75%)	
**BMI at surgery (kg/m** ^2^)		38.9 ± 2.6	38.9 ± 2.6	1.00
**%TWL (%)**		17.9 ± 6.2	NA	NA
**ALT**		30.3 ± 22.5	34.7 ± 23.0	0.48
**AST**		22.9 ± 9.5	26.9 ± 13.3	0.29
**Glucose (mg/dL)**		92 ± 19.1	111.8 ± 35.4	**0.02**
**Insulin (**μ**U/mL)**		24.8 ± 12.3	25.3 ± 14.6	0.92
**HOMA-IR**		5.3 ± 3.0	6.8 ± 3.8	0.25
**Macrovesicular steatosis – n (%)**		14 (58.3%)	23 (95.8%)	**0.004**
**Microvesicular steatosis – n (%)**		3 (12.5%)	21 (87.5%)	**< 0.001**
**Lobular inflammation – n (%)**		13 (54.2%)	19 (79.2%)	0.12
**Portal inflammation – n (%)**		12 (50%)	21 (87.5%)	**0.011**
**Fibrosis – n (%)**		20 (83.3%)	24 (100%)	0.11
**Hepatocellular ballooning – n (%)**		14 (58.3%)	20 (83.3%)	0.11
**Iron overload – n (%)**		14 (58.3%)	10 (41.7%)	0.39

n = number of individuals; Pre-op = pre-operative; WL = weight loss; NA = not applicable; BMI = body mass index; AST = aspartate aminotransferase; ALT = alanine aminotransferase; HOMA-IR = homeostasis model assessment-insulin resistance.

**P values in bold** indicate statistical significance.

Comparison of the distribution of hepatic histopathological variables revealed significantly lower frequencies of macrovesicular steatosis (58.3% versus 95.8%; P = 0.004), microvesicular steatosis (12.5% versus 87.5%; P < 0.001), and portal inflammation (50% versus 87.5%; P = 0.011) in the pre-operative WL group. A complete comparison of the histopathological variables between groups is presented in Table 2.

## DISCUSSION

There is extensive evidence detailing the relationship between WL and improvement of NAFLD and IR.^16–20^ In the current study, significantly lower blood glucose levels were observed in the WL group, indicating a tendency toward better glycemic metabolism in this group. In addition, lower frequencies of macro- and microvesicular steatosis and portal inflammation were observed in the pre-operative WL group.

The main findings of the study were related to the differential impact of WL on specific histopathological aspects of NAFLD in obese individuals. We observed that WL played a significantly more important role in reducing the degree of liver fat content (steatosis) and portal inflammatory activity, but had no detectable effect on lobular inflammation and fibrosis, indicating that some aspects of NAFLD may be more prone to WL interventions than others, which are seemingly more refractory or persistent.

Macrovesicular steatosis is the most evident aspect of NAFLD and is highly responsive to WL through lifestyle interventions or surgery. Excess lipids in hepatic steatosis are mainly neutral lipids such as triglycerides and cholesterol esters. In hepatocytes, neutral lipids are stored in dynamic organelles called lipid droplets.^21^ During the process of food deprivation, cells shift their metabolism from being dependent on glucose to being dependent on mitochondrial fatty acid oxidation.^22^ A study by Promrat et al.,^23^ in which 28 participants with NASH underwent liver biopsy before and after a 48-week intensive lifestyle intervention, demonstrated a significant reduction only in this variable among individuals who lost more than 7% of their baseline weight.

Microvesicular steatosis is a less understood and less common aspect of the NAFLD spectrum, occurring in 10–30% of cases. It is usually associated with mitochondrial dysfunction, leading to the formation of dysfunctional structures called megamitochondria, and is considered an independent risk factor for disease progression, usually appearing in cases in which there is already established fibrosis.^24,25^ In the current study, the prevalence of microvesicular steatosis was considerably higher in individuals with no WL, indicating that the occurrence of this harmful manifestation may be even more frequent in individuals at high risk for more severe NAFLD, such as those with severe obesity. However, the considerable difference observed in the group of individuals undergoing WL indicates that in the face of significant deprivation, the hepatic response to this pattern of fat deposition is also significant. Hwang et al.,^26^ in a study of paired biopsies of nine overweight living liver donors for transplantation who presented with WL, observed a more significant reduction in microvesicular steatosis compared to macrovesicular steatosis, a finding comparable with the current study.

Within the NASH spectrum, portal and lobular inflammation are important markers of inflammatory activity; however, they are not usually coincidental, as demonstrated in the current study. From a mechanistic point of view, this observation implies the existence of distinct immunopathogenic processes that lead to the initiation and maintenance of inflammation in the lobules and portal tracts. Portal inflammation tends to be chronic and is associated with a greater risk of progression to severe forms of NAFLD. Usually, in situations where WL is acute, there are even reports of an increase in the portal: lobular inflammation ratio.^27^ Interestingly, in the current study, the prevalence of portal inflammation was significantly lower in the WL group, while there was no difference between the frequencies of lobular inflammation. Vilar-Gomez et al.,^28^ in a prospective study with paired biopsies in 261 subjects, observed that the degree of WL was independently associated with improvements in all NASH-related histological parameters, including portal inflammation. Conversely, Salman et al.,^29^ in a study of 81 individuals who underwent sleeve gastrectomy, demonstrated a significant response only to lobular inflammation. Similarly, Praveen-Raj et al.^30^ demonstrated complete resolution of lobular inflammation in 12 of 14 individuals at six months after BS. As baseline data concerning the hepatic histological status of individuals undergoing pre-operative WL were not available, it was not possible to fully compare the findings of these studies with those of the present study. Furthermore, the manner in which different surgical procedures lead to the control and resolution of IR and NAFLD goes beyond WL itself, including changes in portal lipid flow, structural and functional changes in incretin secretion and bile acid resorption, in addition to the development of differentiated modulation of satiety at the central level and changes in intestinal microbiota, among several other mechanisms. Therefore, the response to WL obtained surgically or nonsurgically cannot be readily compared.

In the current study, group matching by BMI at the time of evaluation isolated BMI itself as a confounding factor. It was therefore possible to demonstrate that the less severe findings related to macro- and microvesicular steatosis and portal inflammation were more closely related to the recent trajectory of body mass than to BMI itself, a finding that points to a highly dynamic characteristic of NAFLD evolution. Nevertheless, this dynamic aspect of the disease is seemingly less relevant after the initiation of hepatic fibrosis, as the frequency of this abnormality hardly differed between groups. This observation reinforces the greater refractoriness of NASH- associated fibrosis to WL, either surgically induced or not, similar to the findings observed after Roux-en-Y-gastric bypass-induced WL by Kreve et al.^31^ Although fibrosis is an important marker of severity and risk of progression of NAFLD, it is worth mentioning that all cases of fibrosis in this study were, at most, classified as grade 3 (bridging fibrosis), and no participant presented with any evident clinical sign of cirrhosis.

This study has some limitations that need to be considered. The cross-sectional design could reveal associations; however, causal links were not demonstrated. The different origins of the participants (public system *vs.* health insurance plans) likely led to the selection of individuals from different socioeconomic backgrounds, a factor associated with significant dietary, behavior, and occupation-related differences. As such, the joint analysis of the entire sample without stratification by origin should be cautiously interpreted, as it is somewhat biased by this mix-up of individuals from diverse backgrounds. We also identified a relevant gender disparity within both populations, with a remarkable predominance of women despite comparable obesity rates across genders. This likely occurs for various reasons, which encompass, among others, patients’ perceptions of weight-loss surgery, provider referral patterns, patient selection, and sociocultural aspects.^32^ In relation to postoperative outcomes, there is no consensus as to whether gender plays any role. Bal et al.^33^ reported that male gender predicted higher morbidity and lower WL in an analysis of the 2015–2017 data from the American College of Surgeons National Surgical Quality Improvement Program, while Kennedy-Dalby et al.^34^ found no differences in a retrospective matched study. Female predominance could also have influenced the findings of the current study, since NAFLD is usually both more prevalent and severe among men, leading to some degree of underestimation of the disease in our sample.^9,35^ However, the current study has relevant strengths that also merit emphasis. The use of detailed histological analysis to assess NAFLD allowed for an in-depth and nuanced assessment of the differences between groups. Matching by gender, age, and BMI made it possible to isolate relevant confounding variables and analyze WL in isolation, reducing the risk of selection bias and increasing the validity of the observed findings.

The current study’s findings highlight the relevance of liver disease among candidates and patients undergoing BS, emphasizing the necessity of thorough evaluation and careful follow-up of this population in relation to NAFLD, as well as the importance of encouraging individuals to achieve WL and even referring them to BS whenever warranted according to current guidelines. Furthermore, it highlights the importance of liver biopsy collection during BS for several reasons. The operated patient presents with a high risk for NAFLD and its advanced forms, and the proceeding itself is safer in this surgical context; the increase in costs is not prohibitive and, most importantly, the method is the most accurate for these goals. Liver biopsy, in addition to promoting early diagnosis of both NAFLD and severe forms, such as liver cirrhosis, helps to understand the natural evolution of the disease and its postoperative course.^36,37^

## CONCLUSION

Individuals undergoing pre-operative WL presented with significantly lower frequencies of macro- and microvesicular steatosis and portal inflammation, as well as lower blood glucose levels, compared to a group with no pre-operative WL matched by sex, age, and BMI, indicating an association between the recent trajectory of body mass and histological aspects of NAFLD and pointing to the highly dynamic and responsive WL features of this disease.
